# Exploiting the Endogenous Ubiquitin Proteasome System in Targeted Cancer Treatment

**DOI:** 10.3390/cancers15010256

**Published:** 2022-12-30

**Authors:** Noa Hauser, Joud Hirbawi, Meshi Saban Golub, Samar Zabit, Michal Lichtenstein, Haya Lorberboum-Galski

**Affiliations:** Department of Biochemistry and Molecular Biology, Institute for Medical Research Israel-Canada, Faculty of Medicine, The Hebrew University of Jerusalem, Jerusalem 9112102, Israel

**Keywords:** chimeric proteins, Smurf2, IL-2, IL-2R, targeted therapy, HECT E3 ligase

## Abstract

**Simple Summary:**

The design of specific targeting reagents/drugs still remains a major goal in cancer treatment, with the aim of directing therapeutic agents into cancer cells, while avoiding damage to normal tissues and without evoking an immune response. One way to deal with this problem is to use a chimeric protein composed of two components, one to specifically target the cancer cells and the other to kill them. In this study the targeting component is IL2, targeting activated T-cells that over-express the IL2 receptor, and the killing component is Smurf2, a component of the endogenous cellular protein degradation system. When the IL2-Smurf2 chimeric protein enters cancer cells in a dose- and time-dependent manner, it creates a large number of downstream degradation processes, which lead to apoptotic cell death. In this way, the cancer cells program their own death in a non-inflammatory pathway.

**Abstract:**

To overcome the lack of specificity of cancer therapeutics and thus create a more potent and effective treatment, we developed a novel chimeric protein, IL2-Smurf2. Here, we describe the production of this chimeric IL2-Smurf2 protein and its variants, with inactive or over-active killing components. Using Western blots, we demonstrated the chimeric protein’s ability to specifically enter target cells alone. After entering the cells, the protein showed biological activity, causing cell death that was not seen with an inactive variant, and that was shown to be apoptotic. The chimeric protein also proved to be active as an E3 ligase, as demonstrated by testing total ubiquitination levels along with targeted ubiquitination for degradation. Finally, we tested IL2-Smurf2 and its variants in an in vivo mouse model of leukemia and demonstrated its potential as a drug for the targeted treatment of cancer cells. In the course of this work, we established for the first time the feasibility of the use of Smurf2 as a killing component in chimeric targeting proteins. Utilizing the IL2 cytokine to target cells overexpressing IL-2R and Smurf2 to cause protein degradation, we were able to produce a chimeric protein with dual functionality which causes targeted cell death.

## 1. Introduction

Considerable efforts have been invested in recent years in the development of less toxic effective drugs for targeted cancer therapy. Currently, this diverse group of drugs includes monoclonal antibodies, immunomodulators (such as PD-1 or PDL-1 inhibitors), proteasome inhibitors, tyrosine kinase inhibitors, and deacetylation agents [1,2]. Another approach to treating malignancies is the use of chimeric proteins. Chimeric proteins, designed and constructed by gene fusion techniques, comprise both a cell-targeting component and an active component. Over the last few years, we have utilized human pro-apoptotic proteins as the active components of chimeric proteins [3]. Here, we propose for the first time to explore the possibility of using components of the ubiquitin protein degradation system to induce death of target cells. We hypothesize that upon entry of the chimeric protein into cancerous cells, it will induce downstream degradation processes, leading to cell death. In this way, the cancer cells will program their own death in a non-inflammatory pathway. To test our hypothesis, we chose to use the known targeting molecule interleukin 2 (IL-2)**,** a monomeric, secreted glycoprotein known mainly as a T cell growth factor [4].

IL-2 is the natural ligand of the IL2 receptor (IL-2R), which is composed of a complex of three subunits, IL-2Rα (CD25), IL-2Rβ (CD122), and IL-2Rγc (CD132). The high-affinity (Kd = 10^−11^ M) IL-2R contains all three subunits [5]. High IL-2Rα expression, which increases the affinity to IL-2, has been demonstrated in an array of abnormal cells including malignant cells in patients with adult T-cell leukemia, cutaneous T-cell lymphoma, anaplastic large-cell lymphoma, hairy cell B-cell leukemia, and the Reed Sternberg and associated polyclonal T cells in Hodgkin’s disease as well as acute and chronic granulocytic leukemia cells [6,7]. As a targeting component, IL-2 has been proven to be successful in creating specificity to the targeted cell as a result of the high affinity to the IL-2R, which is over-expressed on activated lymphocytes and is involved in many human diseases [8,9].

An example of an IL2-based chimeric protein is the FDA-approved drug Ontak^®^, a fusion molecule of diphtheria toxin domains linked to IL2 (IL2-DT), which due to production issues was discontinued for use in humans. A new version, E7777, which shares an amino acid sequence with IL2-DT, but has improved purity and an increased percentage of active monomer, has received regulatory approval for use in Japan and is in the process of approval by the FDA in the USA [10,11]. Another example of an immunotoxin with FDA approval is Moxetumomab pasudotox-tdfk (LUMOXITI™), an anti CD22 recombinant immunotoxin, for the treatment of hairy cell leukemia [12].

However, a major impediment to the success of such chimeras is the human immune response they elicit, mainly against the bacterial killing component. It is clear that in order to avoid immunogenicity, the killing component has to be of human origin. Therefore, a new generation of chimeric proteins was developed in our lab, mainly composed of human, non-immunogenic toxins, taking advantage of apoptosis-inducing proteins as novel killing components, such as IL2-Bax [13,14]. These targeting molecules are still at the stage of pre-clinical evaluation and meanwhile we are continuing our search for a new human-based killing component, which would cause a natural cell death. As part of this search, we decided to utilize the natural components of the cells’ protein degradation system.

The degradation pathway for many proteins is via the ubiquitin proteasome system (UPS). This system is mostly controlled by a cascade that involves several types of enzymes including E1, ubiquitin activating enzymes; E2, ubiquitin-conjugating enzymes; and E3, ubiquitin–protein ligases [15]. E3 enzymes, also known as ubiquitin ligases, contain substrate-specific recognition domains and catalyze the final step in ubiquitin transfer. The human genome encodes several types of E3 ligases that are mainly characterized by the presence of a HECT (homologous to E6-associated protein C-terminus), a RING (really interesting new gene) or a U-box domain [16]. While RING and U-box E3 ligases act as scaffolding proteins that bring substrates and E2 enzymes together, HECT E3 ligases can directly catalyze the conjugation of the ubiquitin molecule to the target protein [17]. Smad ubiquitylation regulatory factor 2 (Smurf2) is a member of the HECT family of ligases (with a C2-WW-HECT architecture). Smurf2 has 748 amino acids (86 kDa) and is involved in many biological processes in which it controls the stability of several important proteins with central roles in cell-cycle progression, proliferation, metastasis, genomic stability, senescence and more [18]. In addition, Smurf2 has a critical role in the regulation of many signaling pathways, such as transforming growth factor β (TGF-β) and bone morphogenetic protein (BMP) signaling pathways [19,20], as well as the Wnt [21] and RhoA pathways [22]. Smurf2-deficient mice have increased susceptibility to spontaneous tumorigenesis in various tissues including the liver, lung, pituitary and mammary gland [23,24].

In addition, mice deficient in the E3 ubiquitin ligase Smurf2 spontaneously develop B-cell lymphomas that resemble human diffuse large B-cell lymphoma (DLBCL) with molecular features of germinal center or post-germinal center B cells [25]. Smurf2 expression was also significantly decreased in primary human DLBCL samples, and low levels of Smurf2 expression correlated with inferior survival in DLBCL patients [26].

Protein levels of Smurf2 were found to be lower in human lymphoma and breast cancer tissues relative to non-cancer tissues [24]. In a study of prostate cancers, Smurf2 mRNA levels were lower in advanced tumors compared to less advanced organ-confined tumors, suggesting an association of Smurf2 downregulation with tumor progression [27]. The ability of Smurf2 to ubiquitinate and degrade RNF20, a RING-family E3 that controls histone H2B ubiquitination and genome stability, has been implicated in the tumor suppressive role of Smurf2 in a wide spectrum of tumors [18,24]. Smurf2 E3 ubiquitin ligase plays a crucial role in cell differentiation and apoptosis through regulation of the stability of potential substrate molecules, as evidenced by various reports to date [28,29,30].

Components of the ubiquitination system, such as Smurf2, have not yet been utilized or explored for targeted cancer therapy in the form of chimeric proteins, thus represent a novel approach to cancer treatment. Through regulation of a large number of proteins in multiple cellular compartments, and as part of the HECT family, with its intrinsic catalytic activity, Smurf2 functions as a tumor suppressor as described, and as such was chosen as the killing component of the chimeric proteins in this study.

We constructed, expressed and purified a novel IL2-Smurf2 chimeric protein and tested its cell entry and its effect on cell viability, then confirmed its activity as an E3 ligase. In addition, we characterized the cell death induced by IL2-Smurf2 chimeric protein. Furthermore, we produced a variant chimeric protein with an inactive killing component for confirmation that the effects seen were caused by the Smurf2 component of the chimeric protein. We also designed and produced two variants of the chimeric protein with over-active killing moieties to increase the activity. Finally, we tested the effect of the various variants of IL2-Smurf2 chimeric proteins in an in vivo mouse model.

## 2. Materials and Methods

### 2.1. Construction of the Plasmids Encoding IL2-Smurf2 Chimeric Proteins

A plasmid encoding the IL2-Bax sequence, pre-existing in the laboratory [31], was cut with EcoRI and BlpI restriction enzymes to remove the Bax coding sequence. The full length human Smurf2 was generated and amplified by PCR using a Smurf2 clone as a template (clone number: 7939721, GE Healthcare Dharmacon Inc., Lafayette, CO, USA) with the following synthetic oligonucleotide primers covering the whole coding sequence: 5′ AAGGAATTCTCTAACCCCGGAGGC 3′ (sense), 5′ TTATTGCTCAGCTCATTCCA CAGCAAATCCA 3′ (anti-sense). The Smurf2 fragment, cut with the same restriction enzymes, was ligated into the vector fragment using Quick Ligation Kit (New England Biolabs, Ipswich, MA, USA), 3′ to the IL-2 coding sequence, so producing the plasmid encoding the IL2-Smurf2 chimeric protein. The coding sequence of IL2-Smurf2 chimeric protein was confirmed by sequencing analysis.

As a negative control, a plasmid containing a known deactivating mutation (introducing a point mutation at aa 716, from Cys to Ala) [32] in the active site of Smurf2 was designed and generated. First, two PCR reactions were carried out using: (1) the sense primer containing the mutation 5′ AGCCCACACTGCCTTCAATCGAA 3′ and T7 terminator primer; (2) F1 5′ CTGCAGATGATTCTGAACGGCAT 3′ (sense) and the primer containing the point mutation 5′ TTCGATTGAAGGCAGTGTGGGCT 3′ (anti-sense). Second, both PCR products were used as a template for a third PCR reaction using F1 and the T7 terminator primers. The PCR product was cut with EcoRI and BlpI restriction enzymes and ligated into the vector fragment mentioned above containing the IL2 coding sequence. The coding sequence of IL2-Smurf2*716 (termed *716) chimeric protein was confirmed by sequencing analysis and was found to have the sequence coding the Ala residue.

Two more plasmids, Smurf2*547 and *29/30, were produced in a similar process. In IL2-Smurf2*547 (termed *547), a known activating mutation (introducing a point mutation at aa 547, from His to Phe) [32] was generated in the HECT domain of Smurf2 using two PCR steps: (1) the sense primer containing the mutation 5′ GTGTTTTGGACTTTACCTTCTGTGTT 3′ and T7 terminator primer (anti-sense); (2) F1 5′CTGCAGATGATTCTGAACGGCAT 3′ (sense) and the primer containing the point mutation 5′ AACACAGAAGGTAAAGTCCAAAACACC 3′ (anti-sense). In IL2-Smurf2*29/30 (termed *29/30), a known activating mutation (introducing both mutations at aa 29/30, from Phe Phe to Ala Ala) [33] was generated in the C2 domain of Smurf2 using two PCR steps. First, two PCR reactions were carried out using: (1) the sense primer containing the mutation 5′ AAAACCTGGTGAAAAAGGATGCTGCCCGACTTCCT GATCCATTTGC 3′ and T7 terminator primer (anti-sense); (2) F1 5′ CTGCAGATGATTCTGAACGGCAT 3′ (sense) and the primer containing the point mutation 5′ GCAAATGGATCAGGAAGTCGGGCAGCATCCTTTTTCACCAGGTTTT 3′ (anti-sense). For both activating variant plasmids, in the next step both PCR products were used as a template for a third PCR reaction using F1 and the T7 terminator primers. The PCR products were cut and ligated into the vector fragment mentioned above containing the IL2 coding sequence. The coding sequence of each variant chimeric protein was confirmed by sequencing analysis.

### 2.2. Expression and Production of the Chimeric Proteins

Plasmids were transformed into the Rosetta *Escherichia coli* strain, and 0.1 M potassium glutamate and 0.1% glycerol were added when an OD_600_ of 0.2–0.3 was reached. The temperature was then raised to 42 °C for 25 min followed by growth at 37 °C until an OD_600_ of 0.7–0.8 was reached. Protein expression was induced by incubating with 1 mM isopropyl-1-thio-D-galactopyranoside (IPTG), at 16 °C overnight. The cells were then centrifuged for 20 min at 4000× *g*, and the pellet was frozen at −80 °C at least for 30 min. The frozen cells were thawed and suspended in lysis buffer (20 mM Tris-HCl, pH 8.8; 0.05 M NaCl; 0.2 mg/mL lysozyme; 1 mM phenylsulfonyl fluoride (PMSF)) for 30 min at room temperature, followed by sonication. A sample was taken from the lysed cells and marked as whole cell extract (WCE). The remaining cells were then centrifuged at 29,000× *g* for 30 min. The supernatant (marked as soluble fraction; sol) was removed and the pellet (containing inclusion bodies) was suspended in denaturation buffer (6 M urea; 20 mM Tris-HCl, pH 8.8; 0.05 M NaCl) and continuously stirred for 90 min. The solution was cleared by centrifugation at 29,000× *g* for 30 min. The supernatant, containing denatured proteins, was collected and marked as inclusion bodies (IB).

### 2.3. Purification of IL2-Smurf2 Chimeric Proteins

The various IL2-Smurf2 denatured chimeric proteins in 6 M urea (see above) were filtrated and a final concentration of 10 mM imidazole was added. The protein was subjected to immobilized metal affinity chromatography using 5 mL His-Trap columns (GE Healthcare, Sweden) and was eluted with a linear imidazole gradient of 10–500 mM. Fractions containing the chimeric protein were pooled and slowly diluted 1:80 (*v/v*) in refolding buffer (20 mM Tris-HCl, pH 8.8; 2 mM β-mercaptoethanol; 1 mM EDTA; 0.05 M NaCl; 1 mM DTT) and then incubated at 4 °C for 72 h. The refolded chimeric protein solution was then subjected to ion exchange chromatography using a Q-Sepharose column (GE Healthcare, Sweden). Protein was eluted with linear gradient of 0.05–1 M NaCl in refolding buffer. Fractions containing the protein were pooled. Desalting and buffer exchange to phosphate-buffered saline (PBS) was performed using dialysis. The various variant chimeric proteins were aliquoted and kept at −20 °C. All purification procedures were carried out using the FPLC system ÄKTA (GE Healthcare, Sweden).

### 2.4. Characterization of the Chimeric Protein

#### 2.4.1. Structure Prediction

IL2-Smurf2 protein models were generated by AlphaFold v2.0 [34]. Sequences were submitted to the AlphaFold Colab server with the default settings. Each structural model was validated by analyzing the confidence score as generated by the pLDDT (predicted lDDT-Cα). Molecular graphics and superimposition analysis were performed with UCSF ChimeraΧ-1.2.5 [35].

#### 2.4.2. Protein Concentration

Protein concentration was measured according to the Bradford method [36], using Bradford reagent and a standard curve of BSA. Protein concentration was determined at a wavelength of 595 nm.

#### 2.4.3. Separation of Proteins by Gel Electrophoresis

Samples from the various protein sub-cellular fractions (WCE, Sol and IB) were separated on 12% SDS-PAGE gels and stained with Coomassie blue for visualization of the proteins.

#### 2.4.4. Western Blot Analysis

Following separation on SDS-PAGE gel, the proteins were then electro-transferred onto a polyvinylidene fluoride (PVDF) membrane (Bio-Rad, Hercules, CA, USA). The membrane was then blotted with one of the following antibodies: rabbit anti-Smurf2 (1:2000) (SC-25511; Santa Cruz Biotechnology Inc., Dallas, TX, USA), mouse anti-His (1:3000) (27-4710-01; Amersham-Pharmacia Biotech, Uppsala, Sweden), rabbit anti-RNF20 (1:1500) (ab181104; Abcam, Cambridge, UK), mouse anti-FK2 (1:1000) (BML-PW8810; Enzo, Farmingdale NY, USA), rabbit anti-K48 (1:1000) (#8081, Cell Signaling Technology, Danvers, MA, USA), mouse anti-β Actin (1:5000) (A5441; Sigma-Aldrich, St. Louis, MO, USA), mouse anti-tubulin (1:10,000) (ab7291; Abcam, Cambridge, UK), mouse anti-lamin (1:1000) (ab8983; Abcam, Cambridge, UK). The secondary antibodies used were goat anti-rabbit or goat anti-mouse HRP conjugated (1:10,000) (Jackson ImmunoResearch Laboratories Inc., West Grove, PA, USA). Band visualization was performed using the EZ-ECL chemiluminescence detection kit (Biological Industries, Israel).

### 2.5. Cell Lines and Cultures

Mouse lymphocytic leukemia L1210 and human T-cell lymphoma SUDHL-1 cells were grown in RPMI 1640 medium supplemented with 10% heat inactivated fetal bovine serum (HIFBS), 2 mM L-glutamine, 100 units/mL penicillin, 100 µg/mL streptomycin. Karpas, a human non-Hodgkin’s large cell lymphoma cell line, was grown similarly, but supplemented with 10% FBS. Human T-cell lymphoblast YC cells were grown similarly but supplemented with 20% HIFBS. Human T-cell lymphoma HUT102 cells were grown in RPMI 1640 medium supplemented with 10% HIFBS, 2 mM L-glutamine, 100 units/mL penicillin, 100 µg/mL streptomycin, 1 mM sodium pyruvate, 5 mM Hepes buffer solution and 5 µM β-mercaptoethanol. All cells were maintained in flasks and grown in a highly humidified atmosphere of 5% CO_2_ at 37 °C.

### 2.6. Cell Viability Assay

Cells for viability assays were seeded in 96-well plates, 10^4^/100 µL/well and incubated for 1 h. Cells were treated with the chimeric proteins, PBS or a positive control in triplicates and incubated for 24/48/72 h. Cell death was determined using the kit cellTiter-Blue^®^ (Promega, Madison, WI, USA) according to manufacturer’s instructions.

In addition, L1210 cells treated as described above were incubated in the IncuCyte^®^ S3 Live-Cell Analysis System and tracked for up to 72 h. Images of each well were obtained every 2 h. The data were analyzed using IncuCyte^®^ S3 Software 2019A (Sartorius, Gottingen, Germany).

### 2.7. Internalization of the Chimeric Protein into IL-2R Expressing Cells

1 × 10^6^ L1210, HUT102 or SUDHL-1 cells were seeded in 12-well plates and treated with the chimeric proteins (final concentration 15 μg/mL) or PBS (same volume as chimeric protein treatments) and incubated for 24 h. Internalization of the chimeric protein into target cells was examined by Western blot analysis of treated cell extracts using anti- Smurf2 antibodies.

### 2.8. Ubiquitination Asssays in Lysates of IL-2R Expressing Cells

1 × 10^6^ L1210 cells were seeded in 12-well plates and treated with the chimeric proteins or PBS and incubated for 24 h. For testing proteasomal inhibition, additional 1 × 10^6^ L1210 cells were seeded in 12-well plates and treated first with 0.05 µM MG132 and incubated for 24 h, after which they were treated with the chimeric proteins or PBS and incubated for 24 h. Ubiquitination assay was examined by Western blot analysis of treated cell extracts using anti-FK2 antibodies.

### 2.9. Subcellular Fractionation of Target Cells

2.5 × 10^6^ L1210 and HUT102 cells were seeded in 6-well plates and treated with the chimeric proteins or PBS and incubated for 24 h. Subcellular fractionation was performed using the Abcam protocol [37].

### 2.10. In Vitro Caspase3 Activity Assay

L1210 cells (10^4^/100 µL/well in 96-well plates) were treated with the chimeric protein for 48/72 h, and with IL2-Bax and Doxorubicin as positive controls(13,30). Caspase3 activity within the cells was assessed by Apo-ONE^®^ Homogeneous Caspase3/7 Assay Kit (Promega, Madison, WI, USA). Experiments were carried in parallel with cell viability assays. The Caspase3 activity was analyzed using a FLUOstar plate reader at excitation/emission 485/520 nm.

### 2.11. Cell Cycle Analysis by Propidium Iodide Staining

2 × 10^6^ L1210 cells were seeded in 12-well plates and treated with 15 µg/mL IL2-Smurf2 or PBS and incubated for 48 h. Samples were removed for assaying cell viability. Remaining cells (1 × 10^6^ cells) were washed with PBS twice then treated with 1 mL Nicoletti buffer (50 µg/mL propidium iodide, 0.1% sodium citrate, 0.1% Triton X-100). The cells were incubated at 4 °C for 1 h. Cell cycle analysis of the cells was performed using FACScan and the CellQuest program and was analyzed using FCS Express 4.

### 2.12. Real-Time PCR Analysis of Apoptosis-Related mRNAs in Treated Cells

1 × 10^6^ L1210 cells were treated in 12-well plates with 6 µg/mL IL2-Smurf2 or PBS and incubated for 24 or 48 h. Total RNA was extracted from cells using a commercial kit (NucleoBond Xtra Midi, Macherey-Nagel, Düren, Germany). RNA concentrations were determined using a NanoDrop spectrophotometer (Thermo scientific, Waltham, MA, USA). One µg of each mRNA sample was reverse transcribed using a reverse transcription kit (qScript cDNA Synthesis Kit, Quantabio, Beverly, MA, USA).. Individual mRNA levels were quantified using real-time PCR. Each sample contained 2 µL cDNA (20 ng), 1 µL primers (10 ng), 5 µL SYBR^®^ Green and 3 µL H_2_O in a total volume of 10 µL per sample. The primers used are listed in Table 1. The data were analyzed using the primer express program (Applied Biosystems, Foster City, CA, USA).

### 2.13. Mice and Toxicity Studies

Female, 5–6 weeks old DBA/2J SPF mice were obtained from Jackson Laboratory (Bar Harbor, ME, USA). All animal welfare and experimental protocols were approved by the Committee for the Ethics of Animal Experiments of the Hebrew University of Jerusalem (MD-18-15680-5). Mice were housed at a 12 h dark/light cycle and were allowed free access to food and water. This study was carried out in strict accordance with the recommendations in the Guide for the Care and Use of Laboratory Animals of the National Institutes of Health. Adequate measures were taken to minimize pain and suffering. For 10 days, all mice were injected intravenously or intraperitoneally with 15, 30 or 50 µg/mouse/day of IL2-Smurf2. The mice were monitored and weighed 3 times a week.

### 2.14. Induction of Cancer and Experimental Design

A single dose of 10^6^ L1210 mouse lymphocytic leukemia cells was injected intravenously in all mice (day 0). For the following 10 days (days 1–10), the mice were injected with 50 µg/100 µL/mouse/day of IL2-Smurf2, *716, *29/30, *547 or PBS. Following the injection of the proteins or PBS, each mouse was monitored until it reached a predefined humane endpoint. The mice were monitored and weighed 3 times a week.

### 2.15. Statistical Analyses

Results of cells’ viability assays are the average of 3–6 independent biological experiments ± SD. The results of the Western blot analyses were evaluated by densitometry using the Image Lab Software version 5.2.1 (Bio-Rad, Hercules, CA, USA). Statistical analysis was performed using Microsoft Excel 2017 and an unpaired, two-tailed t test was used to determine *p* values. Survival plot and hazard ratio (HR) were visualized and calculated by package “survminer” in R. Pair-wise comparisons were tested with default parameters using the same package [38].

## 3. Results

### 3.1. Construction and Expression of the IL2-Smurf2 Chimeric Protein

**Figure 1:**
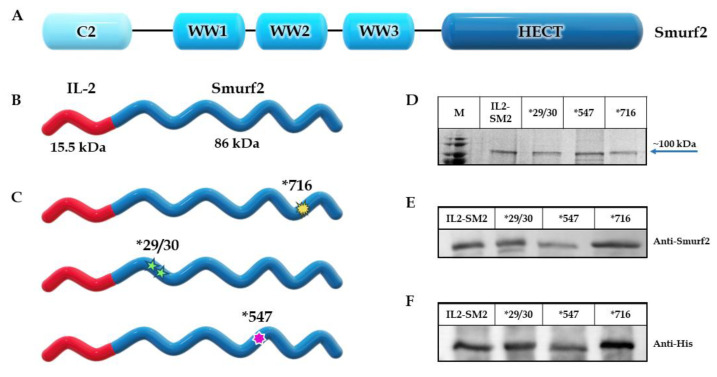
Construction and production of IL2-Smurf2 chimeric proteins: (**A**) Schematic structure of Smurf2; (**B**) Schematic structure of the chimeric protein IL2-Smurf2; corresponding protein size of each component mentioned below the structure; (**C**) Schematic structures of the IL2-Smurf2 chimeric protein variants. Stars marking the point mutations that were introduced in the chimeric protein, in relative location on the Smurf2 moiety for *716 (yellow), *29/30 (green) and *547 (purple) (**D**) Following production, the chimeric proteins were separated on 12% SDS-PAGE and analyzed using Coomassie blue and (**E**) Western blot analysis with anti-Smurf2 and (**F**) anti-His antibodies. Arrows indicate size of the induced chimeric protein. Protein markers on gels are marked by M. The uncropped Western Blot images can be found in Figure S1E,F.

Our aim was to design a chimeric protein able to specifically target and eliminate T cell lymphocytes expressing the IL-2R. As a killing component, we chose to use the human E3 ligase protein Smurf2 (as described in Figure 1A) and as the targeting component, we used the human IL-2 cytokine. The construct consists of the human full-length IL-2 sequence, followed downstream by the human Smurf2 sequence. The sequence encoding the chimeric protein was inserted into a pet28 expression vector under the control of the T7 promotor. Figure 1B illustrates the schematic structure of the final IL2-Smurf2 chimeric protein.

The expression and production were calibrated until the most promising system was established (final protocol described in methods). Following expression, various subcellular fractions—whole cell extract (WCE), soluble fraction (Sol) and inclusion bodies (IB)—were prepared, separated on SDS-PAGE, and characterized both by Coomassie blue staining and by Western blot analyses, using both anti-Smurf2 and anti-His antibodies (Figure S1A,B). Coomassie blue staining revealed major bands with estimated masses corresponding to those of the over-expressed protein: ~100 kDa for IL2-Smurf2, as seen in Figure 1D. Western blot performed with antibodies specific to Smurf2 (Figure 1E) and His (Figure 1F) confirmed the identity of the chimeric protein. These analyses confirmed the successful cloning and production of the in-frame, full-length chimeric protein, in addition to confirmation by sequencing. The three variant chimeric proteins *716, *547 and *29/30 (see methods for the identity of each IL2-Smurf2 variant) were all produced according to the protocol developed for IL2-Smurf2, to minimize the differences between the final chimeric proteins as can be seen in Figure 1C.

### 3.2. Purification of the IL2-Smurf2 Chimeric Protein

Following expression, we found that the protein, being relatively large (~100 kDa), is mostly aggregated in the inclusion bodies (IB) of the bacterial expressing cells (Supplementary Figure S1A,B), and therefore we calibrated and developed a full production protocol that included several steps of purification (see methods for full details). In short, the denatured chimeric protein (in 6 M urea) was loaded onto an affinity column, under denaturing conditions, utilizing the His-tag on the N’ terminus of the protein (Supplementary Figure S1C). Following elution, peak fractions were taken to refolding for 72 h, followed by loading onto an ion exchange column (Supplementary Figure S1D). Peak fractions containing the chimeric protein were dialyzed against PBS. The final chimeric protein preparation was aliquoted and stored at −20 °C.

### 3.3. Structural Prediction of the Chimeric Protein

**Figure 2:**
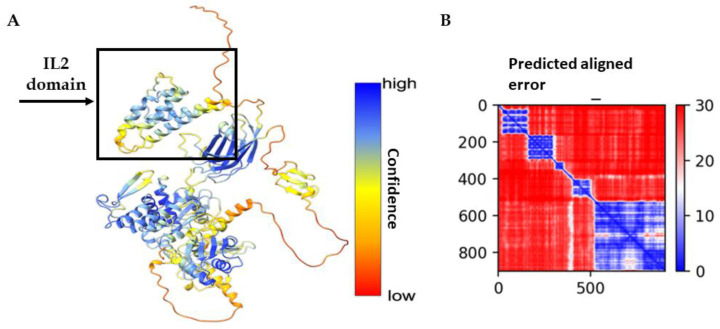
Structure prediction of the chimeric protein IL2-Smurf2: The full-length sequence of IL2-Smurf2 (including the His tag for purification) was entered in the AlphaFold Colab server and was analyzed. (**A**) Ribbon representation of IL2-Smurf2 overall structure. The protein is depicted in colors according to the pLDDT (predicted lDDT-Cα), a per-residue measure of local confidence on a scale from 0–100, where blue is highly confident; (**B**) Predicted Aligned Error (PAE) for the predicted structure. A consistently low PAE at (x, y) (in blue) suggests AlphaFold is confident about the relative domain positions.

The protein sequence of IL2-Smurf2 was examined using the AlphaFold program to obtain a high-level structural prediction. As can be seen in Figure 2A,B, the high confidence in the majority of the structure prediction together with the prediction aligned error (PEA), indicate the high confidence AlphaFold has in the prediction. The most noticeable feature in the structure prediction is the independence of the structure of the IL2 component from the rest of the chimeric protein. This separation of components was not taken for granted, as there is a major size difference between IL-2 and Smurf2 and the smaller component could have been folded inside the larger, thus limiting its function. This prediction is important as the structure of the ligand has a strong effect on the potential ability of the protein to bind to the IL-2R and then efficiently enter the target cell.

### 3.4. Internalization of the Chimeric Proteins into Receptor Expressing Cells

**Figure 3:**
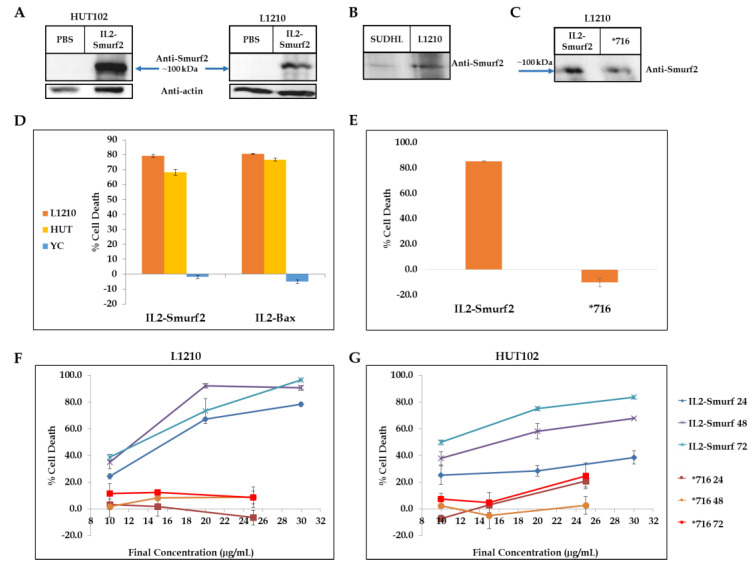
Internalization and biological activity of the chimeric proteins in target cancer cells: (**A**) Western blot analysis with anti-Smurf2 and anti-Actin antibodies of cell lysate after 24 h incubation showing the internalization of IL2-Smurf2 with HUT102 and L1210 cells; (**B**) Under similar conditions we tested the chimeric protein with human SUDHL cells; (**C**) Western blot analysis with anti-Smurf2 of cell lysate after 24 h incubation showing the internalization of IL2-Smurf2 and *716 within L1210 cells; (**D**) L1210, HUT102 and YC cells were treated with IL2-Smurf2 (final concentration: 6 µg/mL) and IL2-Bax (final concentration: 50 µg/mL) for 72 h; (**E**) Cell death of L1210 cells treated with IL2-Smurf2 or *716 for 72 h; (**F**) Cells were treated with various doses of IL2-Smurf2 (shaded blue) or *716 (shaded red) and incubated for 24 h, 48 h or 72 h with L1210 cells (**F**) and HUT102 cells (**G**), showing a dose- and time-dependent cell death. Results are shown as percentage of cell death calculated for treated cells as compared to control cells (treated with PBS only). Results are the average of 3 – 6 repeats ± SD. The uncropped Western Blot images can be found in Figure S2B,C.

To test the internalization of IL2-Smurf2 into IL-2R expressing T cells, L1210, HUT102 and SUDHL-1 cells, known to overexpress the IL-2R, were each incubated with the chimeric protein for 24 h at 37 °C. Cells were thoroughly washed with PBS and then treated with cell lysis buffer. A Western blot analysis of treated cell extracts shows that the chimeric protein is detectable in treated cells, at the expected molecular weight, as seen in Figure 3A,B. Endogenous Smurf2 did not appear in the control cells or in the treated cells (at a lower molecular mass), which may indicate low levels of expression of the Smurf2 protein in these cells, as seen in Figure S2A. After expressing and producing the mutant variant of the chimeric protein, *716, we incubated it with L1210 cells in addition to an active chimeric protein and non-treated cells as control. The mutant *716 chimeric protein entered the cells and appeared at the same band size as the active chimeric protein, as seen in Figure 3C.

### 3.5. Effect of the Chimeric Proteins on Cell Viability and the Specificity of the Effect

To test whether the chimeric protein is biologically active after expression and purification, we examined the effect of the chimeric protein on cell viability. We used L1210, a mouse lymphocyte cell line, and HUT102, a human lymphocyte cell line, as these express high levels of IL-2R. As a positive control for the experiment, we used the previously tested chimeric protein IL2-Bax [31]. All cells were incubated with IL2-Smurf2 (final concentration 6 µg/mL), IL2-Bax (final concentration 50 µg/mL), or PBS (as control; untreated cells) for 72 h. As seen in Figure 3D, the IL2-Smurf2 chimeric protein and the control IL2-Bax chimera both induced cell death in the targeted mouse and human lymphocyte cells.

IL2-Smurf2 chimeric protein proved to be consistent and efficient in causing cell death in target cells, causing between 75–85% cell death. While IL2-Smurf2 and the positive control protein IL2-Bax caused a similar level of cell death, the concentration of IL2-Smurf2 was nearly 8-fold lower, suggesting it may be more potent. In some of the viability assays, the chimeric protein caused even higher levels of cell death than the IL2-Bax chimeric protein positive control.

YC, a human lymphoblast cell line, served as a negative control cell line as it does not express IL-2R and is not responsive to IL-2 based chimeric proteins as seen in previous studies [14]. Treatment with IL2-Smurf2 at concentrations that caused cell death in the targeted cells had no killing effect on YC cells as can be seen in Figure 3D. Treatment of L1210 cells with the mutant *716 chimeric protein did not kill the cells, as seen in Figure 3E, demonstrating that the killing function of the active protein is due to the Smurf2 killing component of the chimeric protein.

When L1210 cells were treated with a range of concentrations of IL2-Smurf2 (10, 20 and 30 µg/mL), or *716 (10, 15 and 25 µg/mL), or with PBS as control, for 24, 48 and 72 h, the chimeric protein caused a linear dose- and time-dependent increase in the percentage of cell death in L1210 cells (Figure 3F) and in HUT102 cells (Figure 3G).

In summary, the chimeric protein IL2-Smurf2 causes concentration and time-dependent cell death in both mouse and human lymphocytes, an effect that is specific since non-target cells lacking the expression of the IL-2R were not affected.

### 3.6. The Killing Component Functions as an E3 Ligase

**Figure 4:**
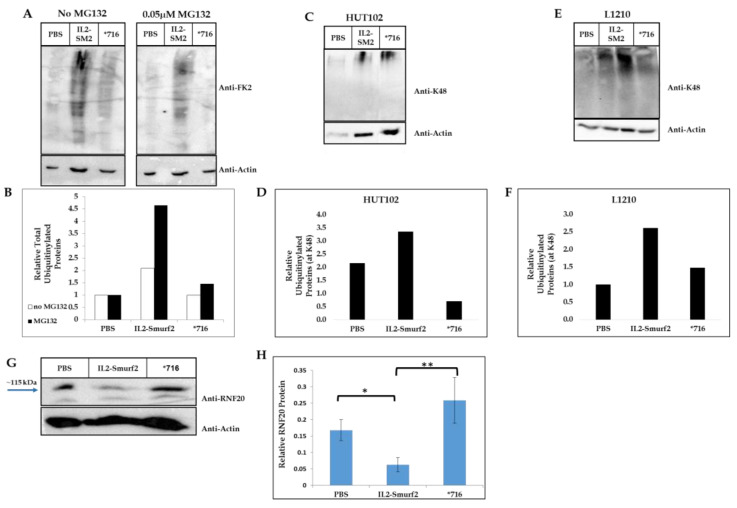
Effect of IL2-Smurf2 and *716 on treated cells: (**A**) L1210 cells were treated with 0.05 µM MG132 or were untreated for 24 h and then 6 µg/mL IL2-Smurf2, 8 µg/mL *716 or PBS was added and the cells incubated for a further 24 h. Western blot analysis was performed with anti-FK2 and anti-β actin antibodies; (**B**) Quantitation of (**A**); (**C**) HUT102 cells were treated with 6 µg/mL IL2-Smurf2, 8 µg/mL *716 or PBS and incubated for 24 h. Western blot analysis was performed with anti-K48 and anti-β actin antibodies; (**D**) Quantitation of (**C**); (**E**) L1210 cells were treated with 6 µg/mL IL2-Smurf2, 8 µg/mL *716 or PBS and incubated for 24 h. Western blot analysis was performed with anti-K48 and anti-β actin antibodies; (**F**) Quantitation of (**E**); (**G**) L1210 cells were treated with 6 µg/mL IL2-Smurf2, 8 µg/mL *716 or PBS and incubated for 24 h. Western blot analysis was performed with anti-RNF20 and anti-β actin antibodies; (**H**). Quantitative of 3 repetitions of **G**. * *p* < 0.03, ** *p* < 0.01. The uncropped Western Blot images can be found in Figure S3.

Having demonstrated that the chimeric protein is able to enter the cells, we wanted to investigate the mechanism of action downstream of cell entry. To do so, we assessed the level of ubiquitinated proteins in the cell lysate after 24 h treatment with the chimeric protein using the FK2 antibody, an anti-ubiquitin antibody (clone FK2), which specifically targets ubiquinated proteins in the cell lysate. As shown in Figure 4A,B, treatment with the chimeric protein resulted in about a 2-fold increase in the amount of ubiquitinated proteins in the cells, while treatment with the inactive chimera had no effect. MG132 is a proteasome inhibitor that competitively inhibits the proteolytic activity of proteasomes so preventing the degradation of target proteins modified with ubiquitin. When cells were treated with MG132 for 24 h and then with the chimeric proteins for an additional 24 h, there was a significant rise (~4.5-fold) in the amount of ubiquitinylated proteins in the cells. The ubiquitin protein has seven lysine residues which can aid in chain elongation and the lysine 48 (K48) is one of the most thoroughly characterized thus far [39]. Proteins marked with poly-ubiquitin chains that contain four or more K48 linked moieties are recognized by the proteosome-mediated degradation signal [40]. When testing the cell lysate for ubiquitinated proteins with K48 linkage, there was a detectable increase in both human and mouse cell lines indicating a 3-fold increase in proteins sent to degradation as a result of the incubation with the chimeric protein (Figure 4C–F). The level of both the general and the K48 specific ubiquitination were similar to control when cells were incubated with the mutant *716 chimeric protein, further supporting the inactivity of this mutant chimeric protein.

In addition, we tested the protein levels of a target protein of Smurf2, Rnf20 [24]. After incubating L1210 cells with IL2-Smurf2, *716 or PBS for 24 h, cell lysates were examined for protein levels of Rnf20 via Western blot, along with actin as loading control. As can be seen in Figure 4G,H, the chimeric protein caused a significant decrease (of ~3-fold) in Rnf20 protein levels while the inactive chimeric protein did not, demonstrating that the killing component can still function as an E3 ligase even when attached to IL2.

### 3.7. IL2-Smurf2 Chimeric Proteins Are Causing Cell Death via Apoptosis

Thus far, we have demonstrated that the IL2-Smurf2 chimeric protein enters the cell and causes a chain of events that leads to cell death in a time- and dose-dependent manner, specifically in target cells expressing the IL-2R. When developing a new approach/reagent for possible cancer treatment it is critical to know how cell death is being caused and therefore we examined various cellular mechanisms that are activated after treatment with the chimeric protein, focusing on the apoptotic pathway.

Caspase3 is not a direct target for Smurf2 and has not shown any clear link to the IL2 cascade. Therefore, we assume that any differences in the cellular Caspase3 enzymatic activity in treated cells compared to the control cells are a result of the cells entering the apoptotic death pathway.

**Figure 5:**
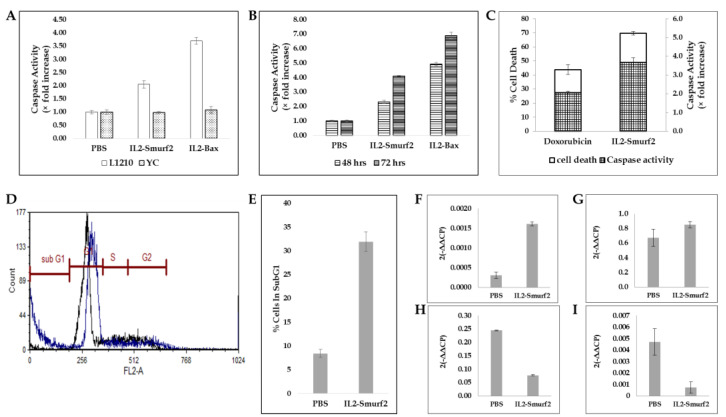
IL2-Smurf2 effect on cell death. (**A**) L1210 and YC cells were treated with 6 µg/mL IL2-Smurf2, 50 µg/mL IL2-Bax or PBS and incubated for 48 h and then Caspase3 enzymatic activity was measured; (**B**) L1210 cells were treated with 6 µg/mL IL2-Smurf2, 50 µg/mL IL2-Bax or PBS and incubated for 48 and 72 h and then Caspase3 enzymatic activity was measured; (**C**) L1210 cells were treated with 6 µg/mL IL2-Smurf2 and 4 µM doxorubicin and then Caspase3 enzymatic activity as well as cell death were measured. Results are shown as fold increase in Caspase3 activity in the treated cells compared to untreated cells. Results are the average of 3 – 6 repeats ± SD; (**D**) L1210 cells were treated with 15 µg/mL IL2-Smurf2 or PBS for 24 h. FACS analysis with the division into cell cycle stages indicated, treated cells are marked in blue, untreated cells (PBS) are marked in black; (**E**) Quantification of total cells in Sub-G1; Real-time PCR results for cellular mRNA mouse Bax (**F**), mouse Bcl2 (**G**) human Bax (**H**) human Bcl2 (**I**) expression levels when treated with IL2-Smurf2 in comparison to untreated cells (PBS). All results represent an average of triplicates.

L1210 cells were incubated with IL2-Smurf2 for 48 or 72 h, and then Caspase3 activity within the cells was measured. As demonstrated in Figure 5A, 48 h treatment with IL2-Smurf2 or IL2-Bax (positive control [13,31]) increased cellular Caspase3 activity in L1210 cells 2-fold and 3.7-fold, respectively. Comparing 48 and 72 h incubation, the increase in Caspase3 activity can be seen to be time-dependent (2.25-fold after 48 h, 4-fold after 72 h; Figure 5B). The increase in cellular Caspase3 activity coincided with an increase in the percent cell death (Figure 5C). IL2-Smurf2 chimeric protein had no effect on Caspase3 activity in non-target YC cells (Figure 5A).

We also investigated the effect of IL2-Smurf2 chimeric protein on cell cycle. L1210 cells were treated with IL2-Smurf2 for 24 h. As can be seen in Figure 5D,E, L1210 cells treated with IL2-Smurf2 had about 32% of cells in sub-G1, which was significantly higher (3.8-fold) than in control cells (8.5%), suggesting that death induced by IL2-Smurf2 is, most probably, via apoptosis.

Next, we tested the effect of the chimeric protein on the expression of apoptosis-related proteins. L1210 and HUT102 cells were treated with IL2-Smurf2 or PBS for 24 h, then total mRNA was extracted and analyzed by real-time PCR for the expression of Bcl2 and Bax, which are anti-apoptotic and pro-apoptotic, respectively. As can be seen in Figure 5F,G, IL2-Smurf2 treatment of L1210 cells resulted in an increase in cellular mRNA levels of pro-apoptotic Bax of 5-fold compared to untreated cells. Cellular mRNA levels of anti-apoptotic Bcl2 were a third of the levels seen in untreated control cells. Similar results were seen in HUT102 cells (Figure 5H–I). The reduction in anti-apoptotic Bcl2 expression even greater in these cells with the untreated cells having six times more Bcl2 than the treated cells.

In summary, we conclude that IL2-Smurf2 chimeric protein causes apoptotic cell death following entry into target cells.

### 3.8. Enhancing Smurf2 Activity Increases Cell Death Caused by the Chimeric Protein

**Figure 6:**
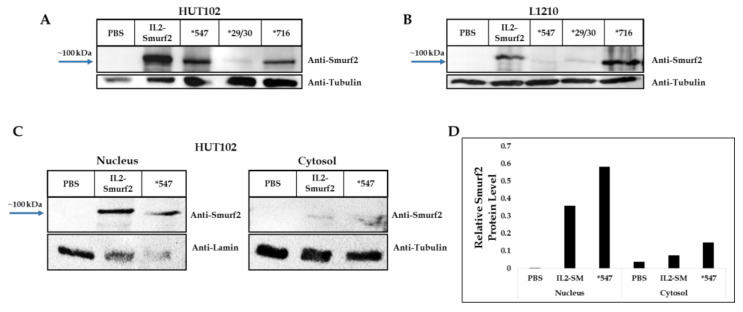
Internalization of IL2-Smurf2 variant chimeric proteins into target cells: (**A**) Western blot analysis with anti-Smurf2 of cell lysate following 24 h incubation showing the internalization of IL2-Smurf2, *29/30, *547 and *716 within human HUT102 cells; (**B**) Under similar conditions, we tested the various chimeric proteins with L1210 cells; (**C**) Following sub-fractionation of cells treated under similar conditions, the nuclear and cytosolic sub-fractions were analyzed using anti-Smurf2 and anti-lamin or anti-tubulin antibodies, respectively; (**D**) Quantitation of (**C**). The uncropped Western Blot images can be found in Figure S4.

Two more IL2-Smurf2 chimeric protein variants were constructed, expressed and produced, *29/30 and *547, respectively containing auto ubiquitination and auto catalysis mutations, (structural representation shown in Figure 1C). Coomassie staining and Western blot demonstrated that the two variants had the same band size as the original chimeric protein (Figure 1D–F). The variants also successfully entered target cells in both HUT102 and L1210 cells (Figure 6A,B). After sub-fractionation, the IL2-Smurf2 protein was found to be located in the nucleus, as was the *547 protein (Figure 6C).

**Figure 7:**
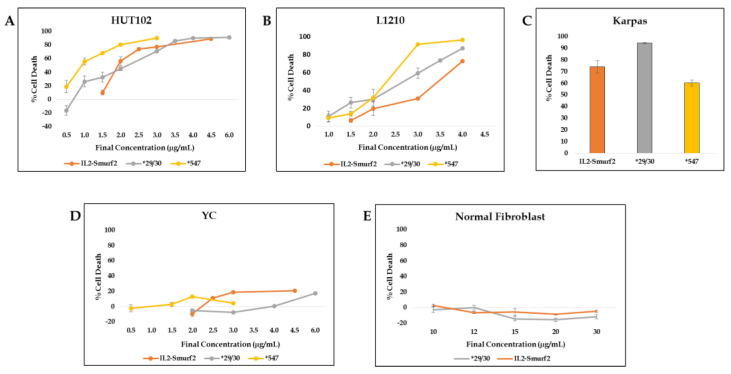
The IL2-Smurf2 variant chimeric proteins effect on cell death: (**A**) HUT102 cells were treated with various doses of IL2-Smurf2 (orange), *547 (yellow) and *29/30 (grey) and incubated for 72 h, showing dose-dependent cell death; (**B**) L1210 cells were treated with various doses of IL2-Smurf2 (orange), *547 (yellow) and *29/30 (grey) and incubated for 72, showing dose-dependent cell death; (**C**) Karpas cells were incubated with IL2-Smurf2, *547 and *29/30; (**D**) YC cells were treated with various doses of IL2-Smurf2 (orange), *547 (yellow) and *29/30 (grey) and incubated for 72 h; (**E**) Normal fibroblasts were treated with various doses of IL2-Smurf2 (orange), *547 (yellow) and *29/30 (grey) and incubated for 72 h. Results are shown as percentage of cell death calculated for treated cells compared to control cells (treated with PBS only). Results are the average of 3 – 6 repeats ± SD.

When HUT102 cells were incubated with chimeric proteins at a range of concentrations, the ‘overactive’ proteins caused cell death at lower concentrations than the original chimeric protein (Figure 7A). While there was little difference in the dose–response curves in L1210 cells, the overactive chimeric proteins did cause more cell death than the original chimeric protein (Figure 7B). In addition, when the human target cell line Karpas, with high expression levels of the IL-2R, was used, *29/30 treatment resulted in a higher percent cell death than the same dose of IL2-Smurf2 (Figure 7C). Increasing the cell killing activity of the chimeric protein might be expected to reduce specificity, but we found that the ‘overactive’ chimeric proteins did not cause significant cell death in the non-IL2R expressing cell line, YC, or in the off-target cell line of normal fibroblasts (Figure 7D,E). Therefore, we can assume that over-activating the Smurf2 component, by increasing the ubiquitination or the catalysis, can improve the killing effect of the chimeric protein, without reducing specificity.

**Figure 8:**
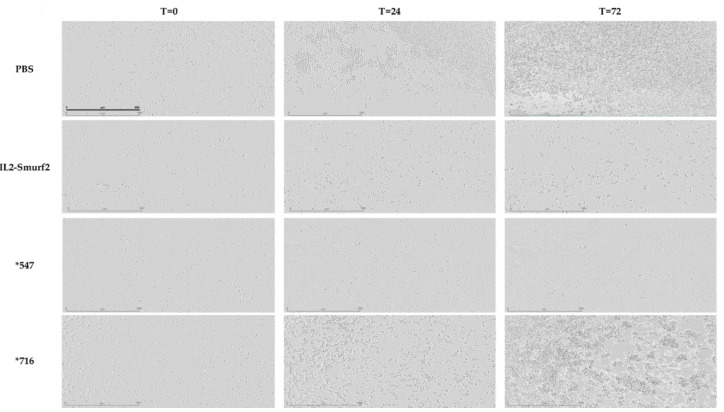
The effect of IL2-Smurf2 on cell death in L1210 cells. L1210 cells were treated with 12.5 µg/ml IL2-Smurf2, *547, *716 or PBS and incubated in the Incucyte machine for 72 h. Microscope images were taken at the beginning (T = 0) and then every 2 h until the end at T = 72 (Magnification 10x).

Similar results were observed when testing the effects of the various chimeric proteins via live imaging. L1210 cells were treated with equal concentrations of 12.5 µg/mL of IL2-Smurf2, *547, *716 or PBS as control, incubated for 72 h and imaged every 2 h using the Incucyte system. As seen in Figure 8, the images at the beginning (T = 0) show similar amounts of viable cells. However, after just 24 h there was noticeable proliferation in the cells treated with PBS and even with *716. In comparison cell death was noticeable in the IL2-Smurf2 and *547-treated cells, an effect which was enhanced in the final images taken at 72 h. Taken together we can conclude that the point mutations in the chimeric protein variants result in higher cell killing activity.

### 3.9. The Effect of the IL2-Smurf2 Chimeric Proteins in an in Vivo Mouse Cancer Model

We based our mouse cancer model on one in which L1210 cells are injected IP into DBA/2J mice [41], however, changing the route of induction to IV was more beneficial in our experiment. We also tested the toxicity of the chimeric proteins on the animals at various concentrations using both IV and IP injections. We monitored the mice to follow any adverse symptoms but witnessed none.

For the experiment, we injected the mice IV with L1210 cells followed by 10 days of IV injections of the treatment. Each group of 5 female mice were treated with one of the following: PBS, 50 µg IL2-Smurf2, 30 µg IL2-Smurf2, or 50 µg *716. During and after the 10 days of injections, we monitored the mice closely for any sign of tumor development as well as general behavior and body weight.

The first group to show signs of distress were the mice treated with the inactive protein, *716. This group reached the final endpoint the quickest, before the control group treated with PBS (Figure S5). The mice in the two groups treated with the different concentrations of IL2-Smurf2 took longer to reach final endpoints and at the end of 40 days both groups had surviving mice with no appearance of external tumors or distress of any kind. These results gave us strong evidence of the proof of concept of the idea that the IL2-Smurf2 chimeric protein could have potential as an effective anti-cancer reagent.

**Figure 9:**
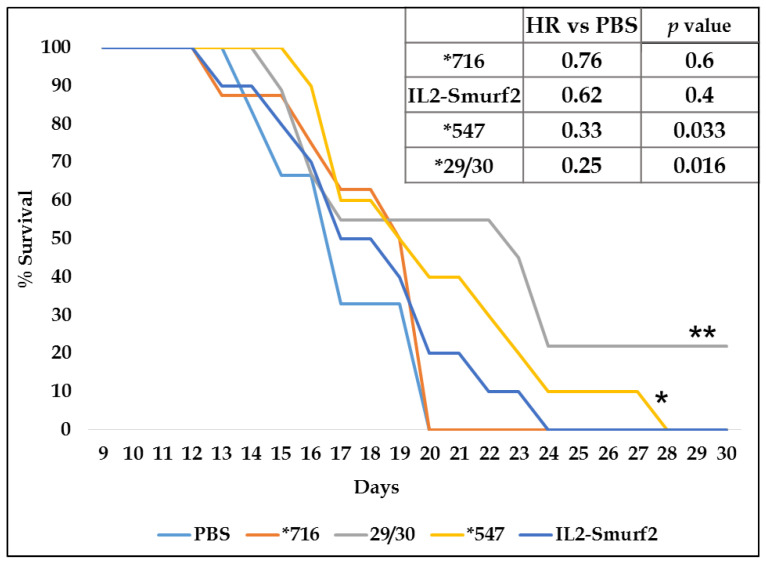
The effect of the IL2-Smurf2 chimeric protein in vivo in a cancer mouse model. Survival of mice treated with 50 µg/100 µL/mouse/day IL2-Smurf2 (N = 10), *716 (N = 8), *29/30 (N = 9), *547 (N = 10), or PBS (N = 7). Hazard ratio of each treatment in comparison to PBS treated mice. * *p* < 0.04, ** *p* < 0.02.

We then carried out a second trial in which we increased the number of mice per group and tested the various IL2-Smurf2 chimeric protein variants. The mice were injected with L1210 cells and then with PBS, IL2-Smurf2, or the variant chimeric proteins *716, *29/30 and *547 (50 µg/100 µL/mouse/day each), and observed daily. Survival in the PBS-treated and the inactive mutant-treated groups was similar and with a high hazard ratio as seen in Figure 9, further strengthening the notion that the killing component is responsible for the cell death. Treatment with the variant overactive chimeric proteins resulted in improved survival, as shown in the survival graph and the lower hazard ratios. The results of this in vivo experiment support the findings of the in vitro experiments whereby the original IL2-Smurf2 has the ability to kill targeted cells and the overactive variants both have a stronger effect. The *29/30-treated group even had surviving mice that were euthanized at the end of the experiment with no obvious endpoints, suggesting possible full recovery.

## 4. Discussion

The development of specific targeting agents remains a major goal in the treatment of human diseases. The hallmark of an effective therapeutic modality is its specificity, the ability to interact with the target cells without undesirable effects in the human recipient. One of the approaches to solve this problem lies in chimeric cytotoxic proteins, whose development has flourished over recent years [42,43]. In cancer research, the goal is to find a killing component that can efficiently kill the targeted cells with high potency but with low levels of damage to the neighboring cells, since it is fused to a precise targeting component.

Earlier developments concentrated on plant or bacterial toxins, which though highly potent, caused severe dose-dependent general toxicity, limiting the maximal dose that could be given to patients. More importantly, the non-human origin of the toxins caused an immune response which resulted in impaired efficacy. Several drugs based on this method were approved by the FDA, for example ONTAK^®^ and Elzonris^®^, and others are currently being researched. The idea of using bacterial toxins for tumor therapy is still attractive but needs further development to achieve better results [44].

It has become clear that the killing moieties should be of human origin, which led our laboratory to the use of human pro-apoptotic proteins as the killing component, hoping that this will prevent an immunogenic response as well as leading to a therapy that is less harmful to healthy cells. However, since these human apoptosis-inducing chimeric proteins are still under development, it is well worth pursuing other directions meanwhile.

The UPS is the main way that cells degrade their proteins. Massive degradation of proteins within cells may lead to cell death. Much research is being conducted on the relationship between different proteins in the UPS and how we can utilize these relationships in drug development [45]. However, the use of a member of the UPS in a chimeric protein has not yet been examined. In this work, we aimed to develop a new chimeric protein prototype based on a protein from the UPS, namely the E3 ligase, Smurf2, as a new “killing” component. Smurf2 specifically has been shown to have an ambivalent role in cell functions [18], but has also been shown to have a connection to cancer, as described in the introduction. We attempted to utilize the UPS in the targeted cells to induce apoptotic cell death. We have described here our approach for developing a chimeric protein that can be used for targeted therapy in hematological cancers.

The chimeric protein IL2-Smurf2 was designed, cloned and expressed in *E. coli* bacteria. When testing the chimeric protein on target cells we found that IL2-Smurf2 is able to target, enter and efficiently kill cells expressing IL-2R, with the ability of the chimeric protein to cause cell death being time- and concentration-dependent.

When studying the mechanism of action of IL2-Smurf2 chimeric proteins after entering the cells, we found that the chimera causes the degradation of proteins specifically targeted by Smurf2, such as RNF20, as well as a general increase in the level of ubiquitinylated proteins. More specifically, when human and mouse cells were incubated with IL2-Smurf2, we saw an increase in the number of proteins marked with ubiquitin and so designated for degradation (by their ubiquitination at lysine 48) (Figure 4C–F). Put together, these results indicate that not only does the targeting component successfully target the cells, but the killing component also shows the expected biological activity; IL2-Smurf2 is active as an E3 ligase and is able to cause massive ubiquitination and degradation of target proteins when entering the target cells, causing finally cell death.

Smurf2 is known to regulate RNF20, whose high expression strongly correlates with tumor formation [46]. As we have shown (Figure 4G,H), the entry of IL2-Smurf2 to the targeted cells caused RNF20 expression levels to significantly decrease, while the mutated chimera did not. This provides evidence that the chimeric protein has tumor suppressor traits in addition to its ability to cause cell death [24].

Remarkably, we found that manipulating the killing component, either by making it inactive or by increasing its activity with point mutations, resulted in a corresponding activity in the targeted cells. While all mutant proteins were expressed and produced in a similar way as the original chimeric protein, and all chimeras were able to enter cells, when the active site cysteine was replaced with alanine, there was no effect on Smurf2 targeted proteins, ubiquitination levels or cell death. Chimeras with increased activity caused greater and quicker cell death. Put together, these results demonstrate that IL2-Smurf2 is active through the cysteine site in the HECT domain in Smurf2. These results further strengthen the notion that the chimeric protein functions as an E3 ligase after entering target cells and so causes mass protein degradation.

The ability of the chimeric protein to function as an E3 ligase indicates that E3 is able to function independently from the UPS cascade, which includes the E1 and E2 enzymes, with its activation raising the total level of ubiquitinated proteins. This phenotype is surprising when considering the high dependency of E3 ligases on the cascade. It is possible that upon entering the target cells in high concentrations, the chimeric proteins activate the other members of the cascade and that way perhaps force activation.

We then studied the nature of the cell death caused by treatment with IL2-Smurf2 chimeric protein. By measuring Caspase3 activity, performing cell cycle analysis and by real-time PCR for apoptotic-related proteins, we found that the chimeric protein, most probably leads to apoptotic cell death, as expected; however, further details are needed. Smurf2 stands out as an E3 that can cause apoptosis itself in downstream reactions, as described earlier [18]. We also hypothesized that a large amount of Smurf2 entering the cell at once would cause massive protein degradation. It may be that Smurf2 has two pathways by which it causes apoptotic cell death—a direct downstream cascade as well as an indirect effect caused by massive degradation. The possibility that Smurf2 has two pathways by which it can cause cell death makes it an even stronger killing component. Originally, Smurf2 was chosen due to its HECT auto-catalyzing traits, which would allow it to cause massive degradation once it enters the targeted cell. However, the choice of Smurf2 has been proven to be beneficial in that in addition, it has a direct pathway that can cause apoptosis.

Finally, we tested the various chimeric proteins in vivo and found that the over-active proteins show a significantly lower hazard ratio, while the inactive chimera had very similar effects as those seen in the control group. We observed that some of the mice in the group that received the mutant *29/30 protein reached the 30th day with no noticeable endpoints, which could indicate full recovery. Many questions remain to be answered, such as the optimization of the concentration and scheduling of treatments and whether this can improve the results. In addition, the stability of the chimeric protein after entering the blood stream and the conditions for optimizing the treatment need to be established. Further understanding of these conditions may highlight the differences between the variant chimeras of IL2-Smurf2.

Immunotherapy, especially CAR T-cell therapy, has demonstrated outstanding response rates in subgroups of patients with hematological malignancies, leading to emergence of CAR T-cells as a breakthrough in cancer immunotherapy [47,48]. Additionally, in recent years, anti-PD therapy has become applicable to a broad spectrum of cancer types, including one clinical trial in Hodgkin’s lymphoma with 87% objective response rates [49]. The efficacy of the therapy depends on many variables including the expression pattern of PD-L1 in the tumor microenvironment, the ability to target tumor-induced immune defects and repair the impaired host immune response [50,51]. While immunotherapy seems to be effective for several malignancies, in some hematological malignancies, response rates are low, and patients still relapse. There also appears to be an issue with the development of drug resistance to some treatments and these therapeutic approaches still need to be optimized in regard to their safety and efficacy. We therefore believe that a combination of such a therapy with our chimeric proteins, in various types of lymphoma and leukemia could potentially perform with higher efficacy, quicker responses and longer survival rates.

It is worth considering conducting a screen to test the combination of IL2-Smurf2 chimeric proteins with small molecular weight drugs, and also to identify enhancing agents to increase its activity, as was conducted for PE-based immunotoxins [52]. Such screening could identify enhancing agents that already have approval for use in the treatment of humans which would increase potency at lower concentrations of both the molecule and the chimera it was given with, thus hopefully reducing any unwanted side effects and shortening treatment duration.

This study has demonstrated the ability to utilize the UPS to suppress tumors and cause-specific cell death, which is a novel and important result. The UPS is endogenous in humans and therefore we expect no or minimal immunogenic response. We have successfully attempted to manipulate this system for the desirable outcome of eliminating cancer cells without compromising their healthy surroundings, a serious problem for many chemotherapeutic drugs. Furthermore, these results may open the door to further investigation of the utilization of E3s for the purpose of causing targeted cell death. Smurf2 is one of the smaller E3s, at ~86 kDa; however, perhaps following the successful shortening of the protein, this can be adapted to other E3s.

In the course of this work, we established for the first time the feasibility of using Smurf2 as a killing component in targeted chimeric proteins. This is the first investigation of a protein from the UPS family connected to a targeting component, and despite previous reports of tumor-enhancing traits of the Smurf2 protein [18], our findings clearly demonstrate the tumor-suppressive activity of the Smurf2 component. The IL2-Smurf2 chimeric protein effectively and specifically caused death by targeting cancer cells, thus opening new approaches in the fight against cancer.

## 5. Conclusions

In this study, we established for the first time the feasibility of using Smurf2, a protein from the UPS family, as a killing domain in chimeric proteins that successfully causes targeted cancer cell death. IL2-Smurf2 has the ability to enter target cancer cells, cause mass protein degradation and thereby lead to apoptotic cell death, while not harming non-target cells. IL2-Smurf2 chimeric proteins may be developed in the future both as a promising targeted cancer therapy and as a treatment for several other pathologies involving uncontrolled expansion of activated T-cells overexpressing the IL2-R.

## Figures and Tables

**Table 1:** Primers used for real-time PCR.

Primer Name	Sense/Antisense	Primer Sequence
mBax (exons 4–5, 130 bp)	sense	AAGCTGAGCGAGTGTTCCGGCG
	antisense	GCCACAAAGATGGTCACTGTCTGCC
mBcl2 (exons 2–3, 151 bp)	sense	CTCGTCCTACCGTCGTGACTTCG
	antisense	CAGATGCCGGTTCAGGTACTCAGTC
hBax (exons 4–5, 116 bp)	sense	TCTGACGGCAACTTCAACTG
	antisense	CAGCCCATGATGGTTCTGA
hBcl2 (exons 2–3, 134 bp)	sense	CCCCTGGTGGACAACATC
	antisense	GGCCGTACAGTTCCCAAA
mGAPDH (exons 5–6, 215 bp)	sense	CCTGGAGAAACCTGCCAAG
	antisense	CAACCTGGTCCTCAGTGTAGC
hG6PD (exons 6–7, 283 bp)	sense	TCTACCGCATCGACCACTACC
	antisense	GCGATGTTGTCCCGGTTC

“m” indicates mice genes and “h” indicates human genes.

## Data Availability

The data presented in this study is available within the article and Supplementary Materials.

## Supplementary Materials

The following supporting information can be downloaded at: https://www.mdpi.com/article/10.3390/cancers15010256/s1, Figure S1: Production and purification of the IL2-Smurf2 chimeric protein; Figure S2: Internalization of the chimeric proteins into target cancer cells. Figure S3: Effect of IL2-Smurf2 and *716 on treated cells. Figure S4: Internalization of IL2-Smurf2 variant chimeric proteins into target cells. Figure S5: The effect of the IL2-Smurf2 chimeric protein in vivo in a mouse cancer model.
