# Supplementary material for: Exploiting the Endogenous Ubiquitin Proteasome System in Targeted Cancer Treatment
Source: Cancers (Basel). 2022 Dec 30;15(1):256. doi: 10.3390/cancers15010256 (PMC9818074; doi:10.3390/cancers15010256)
Supplement: Supplementary file 1 [file cancers-15-00256-s001.zip › cancers-2062603-supplementary.pdf]

Supplementary Materials: Exploiting the Endogenous Ubiquitin Proteasome System in Targeted Cancer Treatment

Noa Hauser, Joud Hirbawi, Meshi Saban Golub, Samar Zabit, Michal Lichtenstein and Haya Lorberboum-Galski

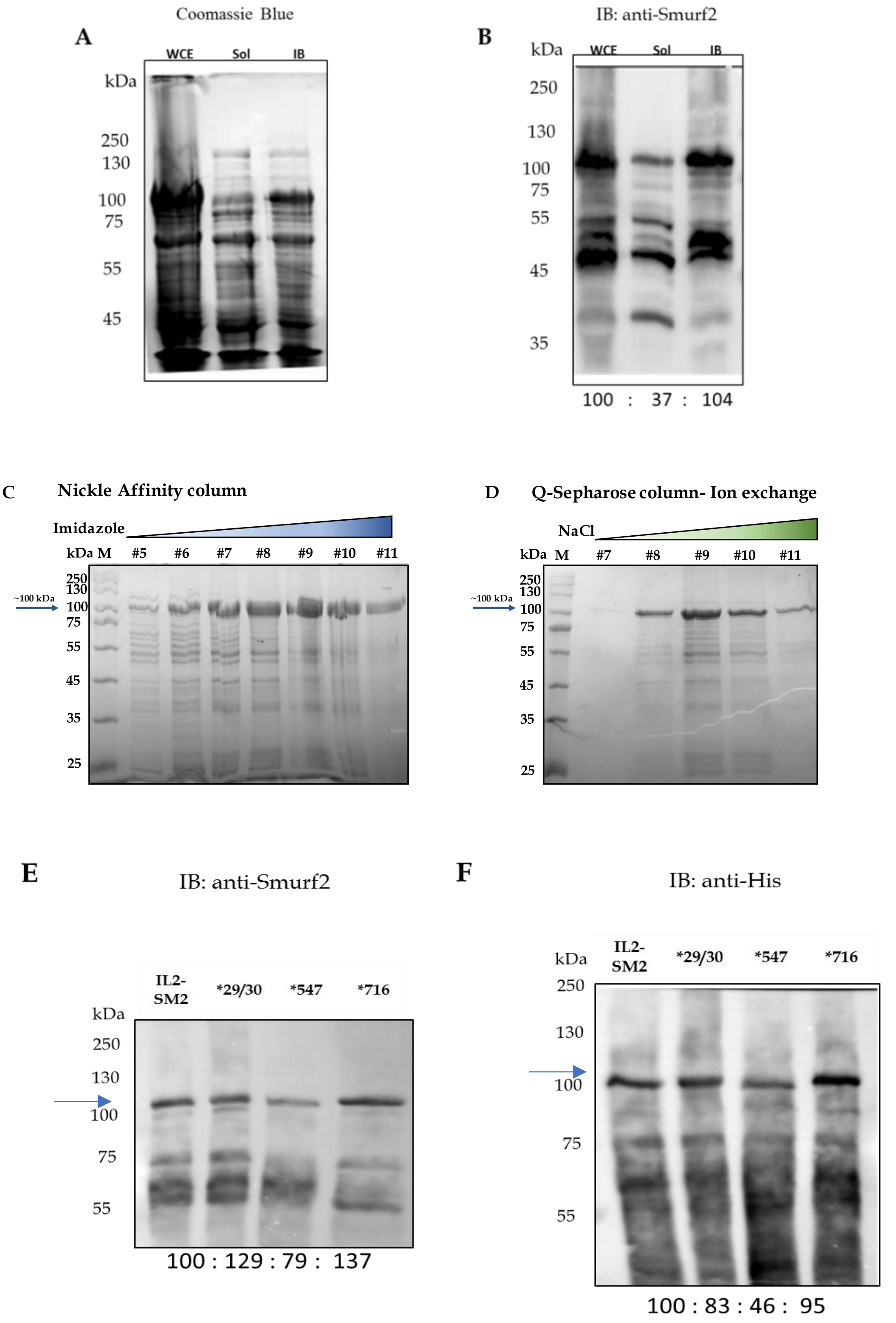

Figure S1. Production and purification of the IL2-Smurf2 chimeric protein: (A) The chimeric protein was expressed in Rosetta strain E. coli. Whole cell extract (WCE), soluble (Sol) and inclusion

bodies (IB) sub-fractions were separated on 12% SDS-PAGE and analyzed using Coomassie blue and (B) Western blot analysis with anti-Smurf2 antibodies; (C) Peak samples from the elution of the chimeric protein on the affinity column; (D) Peak samples from the elution of the chimeric protein on the ion exchange column. All fractions were separated on 12% SDS-PAGE and analyzed using Coomassie blue. Arrows indicate size of the IL2-Smurf2 chimeric protein. Protein markers on gels are marked by M. Following production, the chimeric proteins were separated on 12% SDS-PAGE and Western blot analysis with € (E) anti-Smurf2 and (F) anti-His antibodies was performed. Arrows indicate size of the induced chimeric protein. IB = immunoblotting. Numbers below E&F indicate relative densitometry (to the first value) of the specific band corresponding to the chimeric proteins.

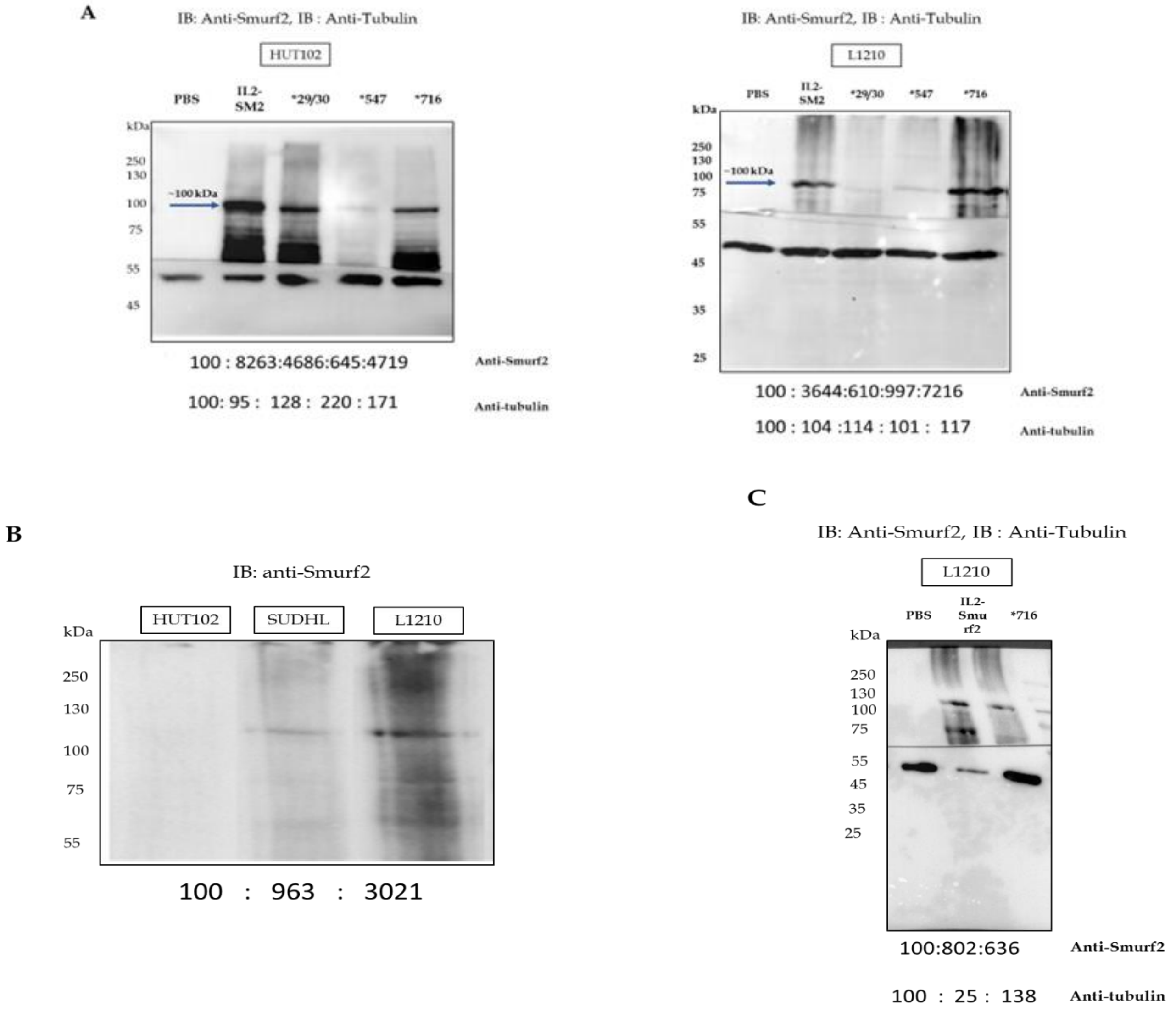

Figure S2. Internalization of the chimeric proteins into target cancer cells: (A) Western blot analysis with anti-Smurf2 antibodies of cell lysate after 24 h incubation showing the internalization of IL2-Smurf2 within human HUT102 and L1210 cells. IB=immunoblotting. Numbers below A (right and left images) indicate relative densitometry to the first value. Internalization of the chimeric proteins in target cancer calls: (B) Under similar conditions (as in Fig. 3A) we tested the chimeric protein with human SUDHL cells; (C) Western blot analysis with anti-Smurf2 of cell lysate after 24 h incubation showing the internalization of IL2-Smurf2 and *716 within mouse L1210 cells. IB=immunoblotting. Numbers below B&C indicate relative densitometry to the first value.

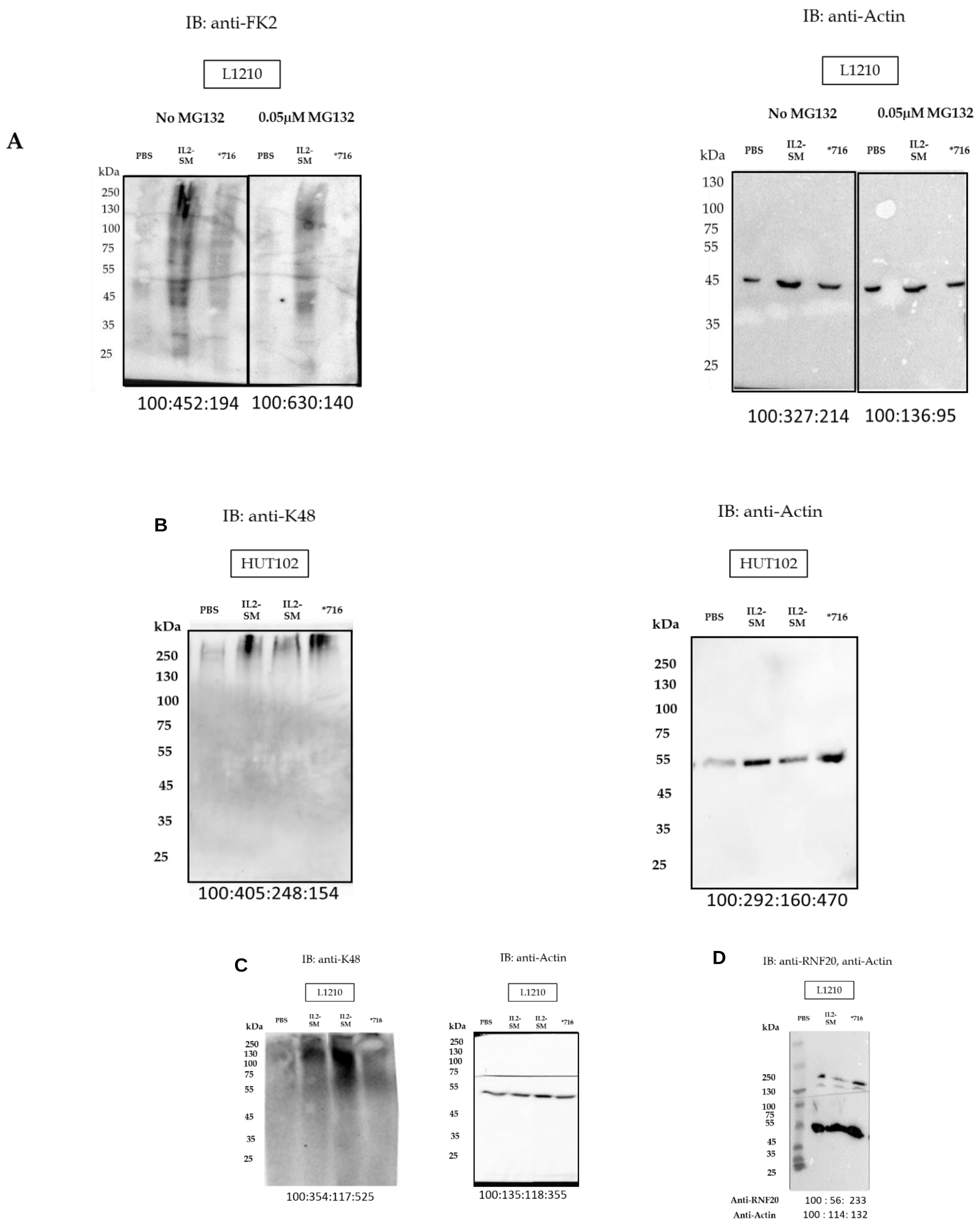

Figure S3. Effect of IL2-Smurf2 and *716 on treated cells: (A) L1210 cells were treated with 0.05 μM MG132 or were untreated for 24 h and then 6 μg/ml IL2-Smurf2, 8 μg/ml *716 or PBS was added and the cells incubated for a further 24 h. Western blot analysis was performed with anti-FK2 and

anti-β actin antibodies. IB = immunoblotting. Numbers below A (right and left images) indicate relative densitometry to the first value. Effect of IL2-Smurf2 and *716 on treated cells: (B) HUT102 cells were treated with 6 μg/ml IL2-Smurf2, 8 μg/ml *716 or PBS and incubated for 24 h. Western blot analysis was performed with anti-K48 and anti-β actin antibodies. IB = immunoblotting. Numbers below images (right and left) indicate relative densitometry to the first value. Effect of IL2-Smurf2 and *716 on treated cells: (C) L1210 cells were treated with 6 μg/ml IL2-Smurf2, 8 μg/ml *716 or PBS and incubated for 24 h. Western blot analysis was performed with anti-K48 and anti-β actin antibodies; (D) L1210 cells were treated with 6 μg/ml IL2-Smurf2, 8 μg/ml *716 or PBS and incubated for 24 h. Western blot analysis was performed with anti-RNF20 and anti-β actin antibodies. IB=immunoblotting. Numbers below images indicate relative densitometry to the first value.

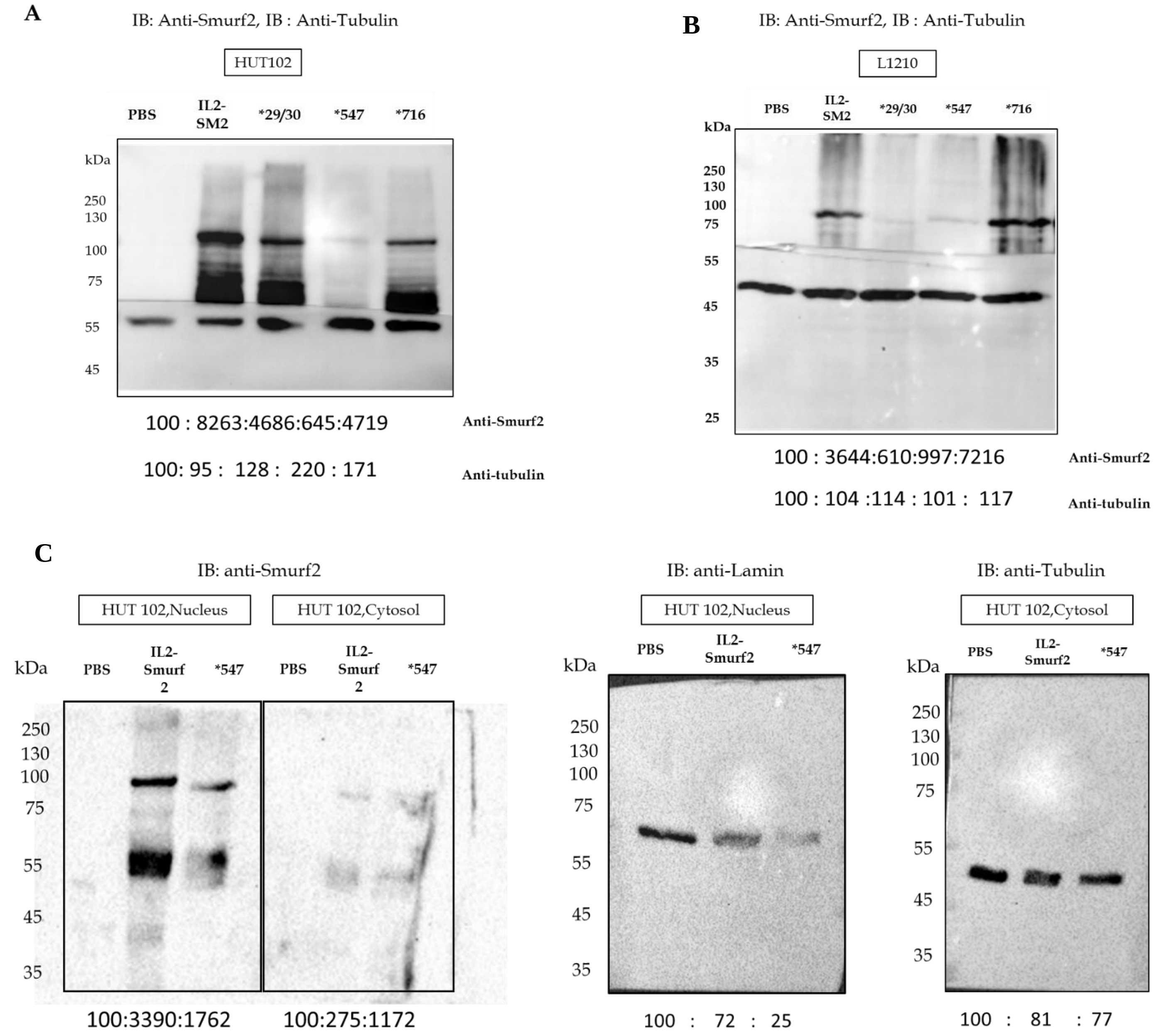

Figure S4. Internalization of IL2-Smurf2 variant chimeric proteins into target cells: (A) Western blot analysis with anti-Smurf2 of cell lysate following 24 h incubation of showing the internalization of IL2-Smurf2, *29/30, *547 and *716 within human HUT102 cells; (B) Under similar conditions, we tested the various chimeric proteins with mouse L1210 cells. IB = immunoblotting. Numbers below images indicate relative densitometry to the first value. In both A&B upper western blot is with anti-smurf2 and lower blot is with anti-tubulin. Internalization of IL2-Smurf2 variant chimeric proteins

into target cells. (**C**) Following sub-fractionation of cells treated under similar conditions as in (A), the nuclear and cytosolic sub-fractions were analyzed using anti-Smurf2 and anti-lamin or anti-tubulin antibodies, respectively. IB=immunoblotting. Numbers below images indicate relative densitometry to the first value.

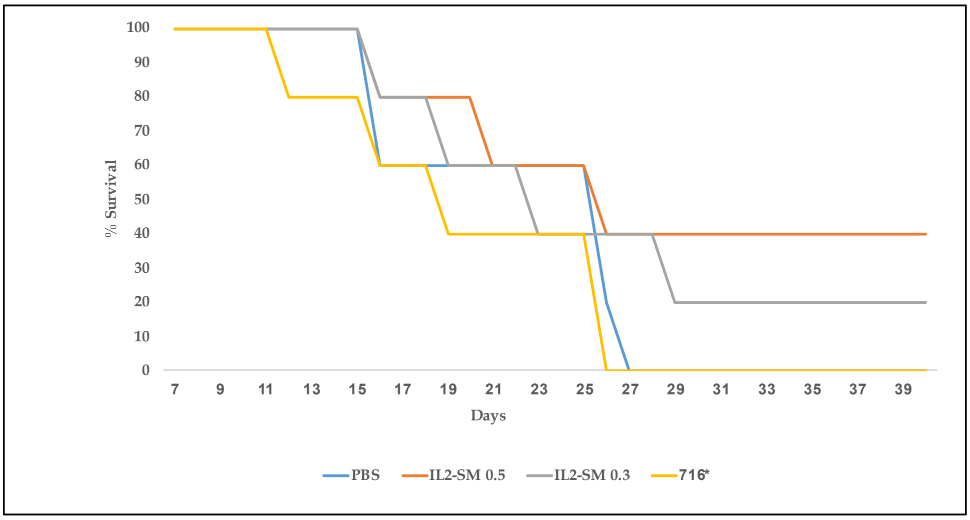

Figure S5. The effect of the IL2-Smurf2 chimeric protein in vivo in a mouse cancer model: Survival of mice treated with 30 or 50 µg/100 µl/mouse/day IL2-Smurf2, 30 µg/100 µl/mouse/day *716 or PBS (*n* = 5).
